# Multi-disciplinary patient-centered model for the expedited provision of costly therapies in community settings: the case of new medication for hepatitis C

**DOI:** 10.1186/s13584-017-0172-1

**Published:** 2017-09-28

**Authors:** Nitzan Avisar, Yael Heller, Clara Weil, Aviva Ben-Baruch, Shani Potesman-Yona, Ran Oren, Gabriel Chodick, Varda Shalev, Nachman Ash

**Affiliations:** 1 Central District, Maccabi Healthcare Services, Yoni Netanyahu 5/31, Petach Tikva, Israel; 2 Medical Division, Maccabi Healthcare Services, Tel Aviv, Israel; 3 Sackler Faculty of Medicine, Tel Aviv University, Tel Aviv, Israel; 4 Institute of Gastroenterology and Liver Diseases, Hadassah Ein Karem Hospital, Jerusalem, Israel

**Keywords:** Hepatitis C virus (HCV), Direct-acting antiviral (DAA) therapy, Health care delivery, Access, Health services management –primary care, Patient-centered care, Community –national health policy, Israel

## Abstract

**Background:**

In January 2015, the first interferon-free direct-acting antiviral (DAA) therapy for chronic hepatitis C virus (HCV) infection was approved for inclusion in Israel’s national basket of health services. During 2015, HCV genotype 1 patients with advanced liver fibrosis (stage F3-F4) were eligible for treatment with ombitasvir/paritaprevir/ritonavir and dasabuvir (OMB/PTV/r + DSV) provided through the four national health plans. As all health plans committed to identifying eligible patients nationwide, risk-sharing agreements created an additional incentive to develop an innovative model for rapid treatment delivery.

**Aim:**

This article aims to describe the development and implementation of a multi-disciplinary patient-centered model for the expedited provision of costly therapies in a community setting, based on experience delivering new HCV therapy in 2015.

**Methods:**

We present the case of the Central District in Maccabi Healthcare Services (MHS), one of five districts in a 2-million-member healthcare provider. We describe the dimensions of the model and its implementation, including the composition and responsibilities of the multi-disciplinary team, screening for patient eligibility, provision of care, and barriers and facilitators identified at each stage.

**Results:**

The experience of the MHS Central District indicates that good communication between all stakeholders was the key driver of successful implementation of the model. Overall, monthly treatment uptake increased following the intervention and by the end of 2015 a total of 99 patients were treated with OMB/PTV/r + DSV in this district. Early data indicate high effectiveness in this population and evaluation in ongoing.

**Conclusions:**

This multi-disciplinary patient-centered model enabled rapid integration of screening and disease staging to identify and treat eligible HCV patients in the MHS central district. The model forms the basis of the 2017 project to deliver DAAs according to broader health basket criteria and may be adapted for the provision of other innovative health technologies in different healthcare settings.

## Background

Hepatitis C virus (HCV) infection is the second leading cause of chronic liver disease worldwide [1, 2]. The ‘post-interferon era’ in HCV treatment [3] has brought on a new paradigm whereby health systems worldwide must develop new strategies to integrate disease screening and staging with access to new therapies [4].

In January 2015, the first interferon-free direct-acting antiviral (DAA) therapy for chronic hepatitis C virus (HCV) infection was approved for inclusion in Israel’s national basket of health services. Each year, new health technologies are reviewed and resource allocation of the upcoming year’s health basket is determined by a multi-disciplinary committee [5]. According to the 2015 health basket criteria, HCV genotype (GT) 1 patients with advanced liver fibrosis (stage F3-F4) were eligible for treatment with ombitasvir/paritaprevir/ritonavir and dasabuvir (OMB/PTV/r + DSV), which was the only HCV DAA approved that year in Israel.

Due to the high cost of this technology and the uncertainty at the time regarding the burden of HCV in Israel and number of eligible patients, introducing DAAs into the Israeli basic health basket with public funding posed a major challenge. Risk-sharing agreements (RSAs) have become increasingly common in the process of updating the health basket Israel in response to budgetary and clinical uncertainty [6]. Introduction of DAAs was made possible financially by establishing a 5-year RSA involving the Ministry of Health, the health plans, and the pharmaceutical company (the first such agreement being with the Israeli branch of AbbVie Inc.). In this model, any expenditure of the health plans beyond a cap was reimbursed by the pharmaceutical company; such that the more patients treated beyond the pre-determined number in the RSA, the greater the potential for health plans to effectively lower the average DAA cost per patient. As all health plans committed to identifying eligible patients nationwide, the RSA created an additional incentive to develop an innovative model for expediting treatment delivery.

Maccabi Healthcare Services (MHS) is the second largest health plan in Israel, with approximately 2 million members across the country. MHS members share similar demographic characteristics to the general population and represent a quarter of the national population (with minor difference related to higher average income and a greater proportion of new immigrants) [7]. In 2012, a total of 10,948 MHS members had a record of HCV infection, the majority of which were immigrants from the Former Soviet Union [8]. HCV GT1 is predominant in Israel [2, 8]. This article describes the experience delivering HCV treatment in the MHS Central district, which is one of five geographical administrative districts and is comprised of 14 sub-districts and 21 branches, with approximately 476,000 members and 192 family physicians in 2015.

Following approval of the first HCV DAA therapy, MHS was faced with the challenge of developing a new strategy for the rapid identification, evaluation and treatment of patients eligible for OMB/PTV/r + DSV. This article describes, retrospectively, the development and implementation of a multi-disciplinary patient-centered model for the expedited provision of costly therapies in community settings, based on experience delivering new HCV therapy in 2015 in the MHS Central District.

## Overview of the model

The stages of the multi-disciplinary patient-centered model and the key stakeholders involved in its implementation are described in Table 1 **.** Potentially eligible HCV patients were first identified using the MHS computerized databases, following which a multi-disciplinary project team was responsible for completing all necessary laboratory tests and disease staging to evaluate eligibility for treatment with OMB/PTV/r + DSV according to the criteria of the national basket of health services: HCV infection confirmed by positive viral load, with genotype 1, and fibrosis stage F3-F4.

**Table 1:** Overview of stages and key stakeholders involved in implementing the model

STAGE	STAKEHOLDER	Action
1. Planning and training	Decision-makers (MHS Central District)	Strategic planning; assigning and training project staff
2. Identifying HCV patients	Expert in data extraction	Identify potentially eligible HCV patients using existing data in the MHS computerized databases
3. Initial communication	Assigned nurse-coordinator (in each sub-district)	Contact local MHS clinics with documented HCV patients; contact managing physicians and patients
4. Laboratory testing	Nurse-coordinator, managing physician	Coordinate referrals to HCV testing and *FibroScan/FibroTest*
5. Eligibility criteria 1: GT1 & F3-F4	Nurse-coordinator	Follow-up laboratory test results and communicate with patients
a. If not eligible	Nurse-coordinator	Refer to gastroenterologist and managing physician for follow-up
b. If eligible	Nurse-coordinator or assigned physician	Set up expedited appointment with assigned gastroenterologist
6. Consultation with gastroenterologist	Gastroenterologist	Evaluate suitability for treatment
7. Eligibility criteria 2: gastroenterologist recommendation	Assigned physician	Follow-up on gastroenterologist recommendations
a. If not eligible	Assigned physician; nurse-coordinator	Refer to gastroenterologist and managing physician for follow-up
b. If eligible	Assigned physician	Contact assigned clinical pharmacist
8. Medication Approval Center	Assigned clinical pharmacist	Submit request for medication approval; coordinate with assigned physician and gastroenterologist to complete any missing information
9. Supply medication to pharmacies	Assigned clinical pharmacist; Central District pharmacist	Ensure supply to pharmacy closest to the patient’s residence; coordinate with AbbVie representatives, with the MHS purchasing department and with pharmacies in each district
10. Patient training in medication use	Assigned clinical pharmacist	After approval and record of first purchase, patients are contacted by telephone and trained in medication use in their mother tongue
11. Regular patient follow-up	Gastroenterologist and family physician	Continued follow-up of patient during and after treatment

## Implementation: Barriers and facilitators

### Strategic planning and training of dedicated healthcare professionals

**Fig. 1:**
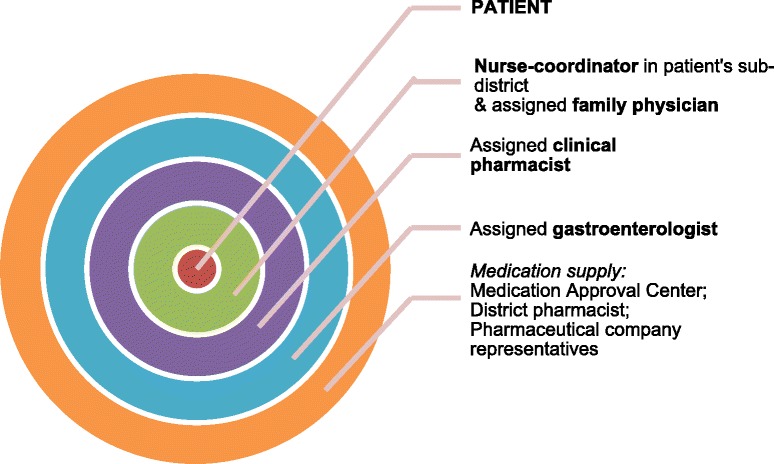
Patient-centered model: composition of the multi-disciplinary project team

The MHS Central District developed a patient-centered model to ensure that a multi-disciplinary team of dedicated healthcare professionals would be available locally to respond to the patients’ needs (Fig. 1). The cornerstone of the model is good communication with the patient, family physician and among the project team, with a central role for assigned nurses and clinical pharmacists in facilitating communication at key stages of the process. The model was initiated in August 2015 and comprised an iterative component and was evaluated on a daily basis to address any challenges encountered during implementation (Table 1).

Successful implementation of the project required strategic planning by the Central District and clearly defined allocation of resources to the project. Representatives from 14 sub-districts were recruited to this unique project and trained in the offices of the Central District. From each sub-district, one or two nurses were assigned to the project, each one for about 1–2 h per day, with 18 nurses in total. The nurses were responsible for coordinating the treatment of hepatitis C patients in their geographical area. This central role of the nurse-coordinator was crucial in establishing and maintaining personal contact with patients.

The training consisted of educational material on HCV, epidemiology, available treatments and current knowledge on efficacy and potential side effects of the new therapies. In addition, nurse-coordinators were trained in methods for accurate data collection and monitoring. At the end of the training course, participants were given access to the HCV patient database in order to view and update patient records. Medical managers in each sub-district also participated in the training.

### Identifying HCV patients

A first data query was performed to identify patients who met any of the following criteria: (i) a diagnosis code (International Classification of Disease, 9th edition, clinical modification) for Hepatitis C; (ii) record of a positive viral load (by polymerase chain reaction, PCR); (iii) a valid HCV genotype test indicating genotype 1; or (iv) a positive HCV antibody test. A total of 956 patients met these criteria. All available data relating to HCV laboratory tests, liver fibrosis and geographical area were entered into a patient database for exclusive use by the trained project staff. Nurse-coordinators reviewed the electronic medical records of each HCV patient in their sub-district in order to ensure the accuracy of the data relating to screening and disease staging. This first stage of selection using the databases was used to exclude patients who did not meet the eligibility criteria due to a recorded genotype GT2, GT3 or GT4 or a recently measured fibrosis stage below F3.

### Initial communication and coordination

Patients who did not meet the initial eligibility criteria in Stage 2 were directly contacted by the assigned nurse-coordinator in the patient’s sub-district, with the recommendation to refer to their managing physician for continued disease management. Patients who were determined to be potentially eligible for treatment were contacted by phone to provide them with key information on treatment options. The nurse-coordinator informed the patients of the new therapy and explained the steps needed to assess their eligibility, including abdominal ultrasound and laboratory tests. Patients were instructed to contact their family physician in order to complete laboratory tests necessary for screening and disease staging. In parallel, the nurse-coordinators contacted the patients’ physician directly to request that the patient be referred for the relevant laboratory tests.

At this stage in the process, the nurse-coordinators received important feedback from physicians which flagged potential challenges in patient outreach and participation of physicians, including: (i) several patients had not encountered the health system in a long time and were not known to any primary care physicians in their community at the time; (ii) in particular, injecting drug users (IDU) would be very hard to reach; (iii) poor availability of primary care physicians and heavy work load in the community may burden certain physicians; and (iv) primary care physicians may be reluctant to engage in the project due to limited familiarity with HCV testing and fibrosis staging.

An assigned physician was recruited to work full-time for a period of three months to help resolve these issues: to coordinate between the team members and all stakeholders involved, to address any challenges encountered, including medical questions and technical issues, and to improve the overall process ensuring that patients rapidly move onto the next stage of evaluation. Assistance was provided by the local secretary, nurses and medical manager to localize patients who could not be reached by phone. Primary care physician who were not available due to the heavy work load or were reluctant to engage in the project due to limited familiarity with HCV testing and fibrosis staging, could use the hepatitis C project team to communicate with the patients and provide laboratory testing. In addition, the process was improved by ongoing communication with nurse-coordinators, consultations with the assigned physician (and with the district management or to a gastroenterologist where necessary).

### Laboratory testing

HCV viral load and genotype testing by real-time PCR were performed on a daily basis in the MHS Central Laboratory to confirm patients’ eligibility.

Fibrosis stage was assessed by two alternative methods: FibroScan and FibroTest. FibroScan is special ultrasound examination performed in hospitals and its results are not automatically shared with MHS. Therefore, the need arose for the MHS HCV project team to collaborate with various medical centers in order to receive results of FibroScan tests previously performed by potentially eligible HCV patients in MHS and update the HCV patient database. FibroTests (based on blood tests) were approved for HCV patients who had already confirmed GT1 and positive viral load and performed in an external laboratory in Jerusalem. Several challenges were overcome at this stage. At the start of the project, FibroTest services were available to MHS twice a week, but this created a significant delay in testing as well as constituting a major access barrier for patients who were not able to go to the laboratory on those specific days. The project team contacted relevant officials and decision-makers and the frequency of testing was increased such that samples were transported to the FibroTests laboratory on a daily basis. MHS laboratory personnel were trained in taking and storing the blood sample until transportation. In addition, a mechanism was set up to reach patients confined to their homes, in order to extend access to FibroTests to all eligible patients.

### Selection of patients with GT1 and advanced fibrosis

The nurse-coordinators were responsible for following after fibrosis staging and contacting patients. Patients with fibrosis stage F0, F1 or F2, who were not eligible to receive the new treatment according to the 2015 health basket criteria were referred to a gastroenterologist to pursue follow-up and discuss alternative options for disease management. Patients with GT1 with fibrosis F3-F4 proceeded to the next stage of the process.

### Consultation with a gastroenterologist

At this stage, eligible patients were referred to a gastroenterologist (facilitated by the nurse-coordinator or assigned physician). The waiting time for an appointment to a gastroenterologist may take between several weeks to months and this was a major bottleneck in the delivery of care. The management of MHS decided to offer financial incentives to gastroenterologists in the Central District and neighboring districts to ensure that these potentially eligible HCV patients had access to a specialist within 1–2 weeks. MHS’ gastroenterology expert was involved in advising the participating gastroenterologists where necessary to determine eligibility for treatment.

### Recommendation for treatment

Upon gastroenterologist recommendation, the assigned project physician promptly contacted an assigned clinical pharmacist to ensure that the medication request (including the relevant test results and gastroenterologist’s recommendation letter) was submitted to the Medication Approval Center.

### Medication approval center

The clinical pharmacist gathered the necessary documentation for pre-authorization and submitted the request for medication approval. The assigned clinical pharmacist then contacted the pharmacist in the Medication Approval Center in order to request expedited approval within hours.

### Supplying medication to local pharmacies

Once the medication approval was obtained, the district pharmacist and clinical pharmacist were responsible for supplying the medication to the pharmacy closest to the patient’s home. There was a need to coordinate this supply with representatives of the pharmaceutical company, with the MHS purchasing department and with the pharmacies in each district to ensure easy access for the patient. Occasionally it was necessary to transfer medication supply between pharmacies and to the periphery, such as the southern city of Eilat.

### Training patients in medication use

Using the computerized databases, the dedicated clinical pharmacists were able to track medication purchases and contact the patient directly after their first purchase. Clinical pharmacist provided guidance over the phone on how to use the medication: frequency and times of ingestion, interactions with other drugs, explanations on potential adverse events and an overview of the follow-up and testing throughout the treatment. These telephone-training sessions were conducted in the patients’ mother tongue and each patient was given the clinical pharmacist’s phone number and encouraged to contact them with any questions.

### Continued follow-up of treated patients

Although the project closed as planned at the end of the year 2015, it forged a strong network for cooperation among healthcare professionals to promote HCV diagnosis and care. In each sub-district there is a nurse who was trained in this project and remains a key point of contact and valuable asset for managing HCV patients. The project team members, assigned physician and clinical pharmacologist are known to have participated in the project and continue to be available to share their knowledge experience in the field. After completion of the project, continued follow-up of patients was provided by family physicians, gastroenterologists, and local nurses.

## Discussion

**Fig. 2:**
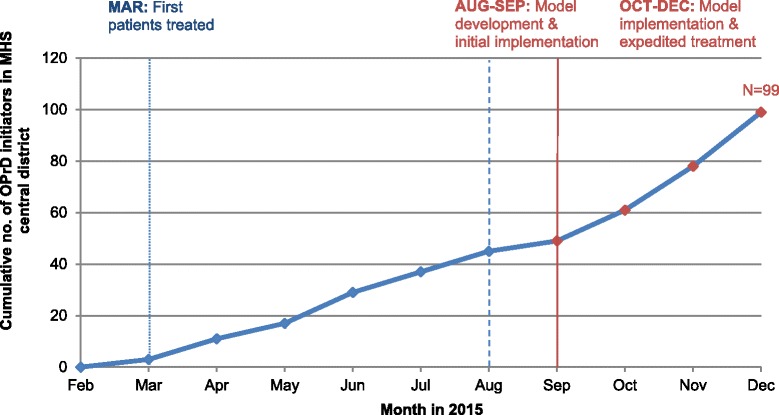
Uptake of OMB/PTV/r + DSV in the MHS Central District in 2015 before and after implementation of the patient-centered model

Communication between decision-makers, healthcare professionals and patients was essential to the successful implementation of the model. Overall, 99 patients in the district were approved for treatment with OMB/PTV/r + DSV in 2015 (Fig. 2), and the dedicated project team ensured follow-up to monitor safety and effectiveness.

The structure of the model enables challenges raised by key stakeholders to be addressed in a timely manner to improve implementation in real-world settings. This ability to be responsive and to adapt to stakeholders’ needs is made possible by open channels of communication between patients, healthcare professions, and decision-makers.

Previous qualitative studies have shown that brief educational interventions can substantially improve knowledge and acceptability of HCV testing and care [9]. Experience with HCV therapy in the MHS Central district suggests that successful implementation of this type of model depends on understanding knowledge gaps and attitudes about the disease and health technologies among patients and all key stakeholders. There is also a need to promote efforts to target hard-to-reach populations, such as drug users [10] and other marginalized populations [11], who may face barriers to access in many countries and in diverse health systems.

This case study underscores the important role of primary care in the delivery of new health technologies. The multi-disciplinary patient-centered model for integrating screening and disease staging to rapidly identify and treat patients may be adapted for the provision of other innovative medicines in Israel and in different healthcare settings. For example, limited epidemiological data are available on non-alcoholic fatty liver disease [12] and a changing treatment landscape will require similar large-scale, multi-stakeholder public health interventions to identify patients and rapidly deliver new interventions in the community setting. In the case of HCV, this model and the lessons learnt from its implementation may be applied in similar settings, as the HCV treatment landscape continues to evolve and screening efforts will need to expand to provide access to a wider patient population.

We would like to emphasize the critical role of RSAs in the adoption of new health technologies. Whereas in 2011 3% of the annual budget allocated for new technologies was subject to RSAs, this proportion rose to 37% in 2016 [6]. At the health plan level, a major challenge remains in ensuring adequate incentives for the continued rapid delivery of innovative medicines, beyond the RSA’s incentives which may be limited to the phase of initial uptake. Our model and the experience described here represent a long-term investment in creating efficient channels of communication, networks of trained healthcare providers, and sustainable tools for tackling similar challenges in the future. With the expansion of the health basket in 2016 and 2017 to include other DAAs and broader indications, new processes based on the 2015 model are considerably facilitated by harnessing the existing knowledge-base and networks of experts. This case study underscores the importance of knowledge sharing and good communication between key stakeholders to tackle uncertainty and promote access to new DAAs, with sustainable solutions to increase awareness and improve implementation in the long term.

Nonetheless, with less favorable RSAs in 2016 and 2017, important opportunities for reaching out to eligible HCV patients may be missed due to limited resources of the health plans. Compared to 2015, there was a decline in the number of patients with genotype 1 F3-F4 initiating any DAA in the MHS central district in 2016. The lower uptake is likely attributable in part to the diminishing pool of eligible patients in the district once most patients meeting these criteria had been identified and treated in 2015, and this rationale contributed to the 2017 health basket decision-making process to expand access to DAAs to other genotypes and fibrosis F2. However, the decline in the number of treated patients may reflect changes in the RSAs, and this experience underscores the potential repercussions on health plans’ incentives for reaching out to high-risk groups given the need to prioritize resource allocation.

In 2015, HCV DAAs were included into the WHO Essential Medicines List as a result of demonstrating unprecedented high SVR rate [13]. In a study of early users of OMB/PTV/r + DSV in MHS in 2015, SVR rates similar to clinical trials (>90%) were observed in the real-world setting, including among patients with cirrhosis and comorbidities [14]. In this respect, the experience of MHS with this model of HCV treatment delivery will continue to be evaluated in parallel to real-world HCV patient outcomes in order to monitor long-term outcomes, inform data-driven decisions, and improve knowledge transfer and exchange [15]. Currently, patients who do not meet eligibility criteria of the national basket of health services may receive DAA therapy through complementary insurance with the health plans, and while these different pathways exist the very small number of patients taking advantage of this option indicates that it is important to apply a similar model to ensure equitable access and quality of care for all patients.

## Conclusions

As an early adopter of new technologies, the Israeli health system faces the challenge of ensuring good access and delivery of care. The multi-disciplinary patient-centered model presented here, with a focus on primary care, provides a unique framework for managing the expedited provision of costly therapies in community settings. The model may be adapted for the provision of other innovative medicines in Israel and in different healthcare settings.

## Abbreviations

DAA: Direct-acting antiviral
GT: Genotype
HCV: Hepatitis C virus
MHS: Maccabi healthcare services
OMB/PTV/r + DSV: Ombitasvir/paritaprevir/ritonavir and dasabuvir
